# Evaluation of intravenous infusion of labetalol versus magnesium sulfate on cerebral hemodynamics of preeclampsia patients with severe features using transcranial doppler

**DOI:** 10.1007/s10877-023-01006-4

**Published:** 2023-04-19

**Authors:** Sherif M. S. Mowafy, Marwa M. Medhat

**Affiliations:** Department of Anesthesia and Surgical Intensive Care, Faculty of Medicine, Zagazig University, Zagazig, Egypt

**Keywords:** Labetalol, Magnesium Sulfate, Cerebral hemodynamics, Severe preeclampsia

## Abstract

**Purpose:**

It is essential to understand the underlying pathophysiological mechanisms of preeclampsia cerebral complications. This study aimed to compare the cerebral hemodynamic effects of magnesium sulfate (MgSO4) and labetalol in pre-eclampsia patients with severe features.

**Methods:**

Singleton pregnant women who suffered from late onset preeclampsia with severe features were enrolled and subjected to baseline Transcranial doppler (TCD) evaluation and then randomly assigned to either the magnesium sulfate group or labetalol group. TCD to measure middle cerebral artery (MCA) blood flow indices including mean flow velocity (cm/s), mean end-diastolic velocity (DIAS), and pulsatility index (PI) and to estimate CPP and MCA velocity were performed as basal measurements before study drug administration and at post-treatment one and six hours after administration. The occurrence of seizures and any adverse effects were recorded for each group.

**Results:**

Sixty preeclampsia patients with severe features were included and randomly allocated into two equal groups. In group M the PI was 0.77 ± 0.04 at baseline versus 0.66 ± 0.05 at 1hour and 0.66 ± 0.05 at 6 hours after MgSO4 administration (p value < 0.001) also the calculated CPP was significantly decreased from 103.3 ± 12.7mmHg to 87.8 ± 10.6mmHg and 89.8 ± 10.9mmHg (p value < 0.001) at 1 and 6 hours respectively. Similarly, in group L the PI was significantly decreased from 0.77 ± 0.05 at baseline to 0.67 ± 0.05 and 0.67 ± 0.06 at 1 and 6 hours (p value < 0.001) after labetalol administration. Moreover, the calculated CPP was significantly decreased from 103.6 ± 12.6 mmHg to 86.2 ± 13.02mmHg at 1 hour and to 83.7 ± 14.6mmHg at 6 hours (p value < 0.001). In terms of changes in blood pressure and the heart rate, they were significantly lower in the labetalol group.

**Conclusion:**

Both magnesium sulfate and labetalol reduce CPP while maintaining cerebral blood flow (CBF) in preeclampsia patients with severe features.

**Trial registration:**

The institutional review board of the Faculty of Medicine, Zagazig University approved this study with the reference number (ZU-IRB#: 6353-23-3-2020) and it was registered at clinicaltrials.gov (NCT04539379).

## Introduction

Preeclampsia is a multi-system disorder of pregnancy characterized by widespread vascular dysfunction. One of the most prominent organs to be affected is the brain. Indeed, central nervous system (CNS) manifestations such as headache, visual disturbances, alterations in consciousness, and seizures are considered severe features of the disorder and significantly contribute to maternal morbidity and mortality [1–3]. Many studies have shown that cerebral vasospasm, hypertensive encephalopathy, excitation of brain receptors, and a hyperactive sympathetic nervous system are all implicated in the unidentified etiology and pathogenesis of eclamptic convulsions, and most of the data suggest hypertensive encephalopathy is a serious consideration in most of the severe preeclamptic patients, and a very small minority of these patients are under perfused (ischemic) [4–6]. As a result, controlling blood pressure while also controlling cerebral perfusion pressure (CPP) is critical in preeclampsia management, and these patients will likely benefit from a medication that primarily induces peripheral vasodilation and reduces CPP while maintaining cerebral blood flow within the normal range [7].

Magnesium sulfate (MgSO4) has been used for seizures prevention in severe preeclampsia for many years with demonstrated efficacy in eclampsia prevention and treatment [8–10]. Its use was reported to be associated with a significant reduction in maternal mortality and nowadays it is considered the standard of care for seizures prophylaxis in severe preeclampsia [1]. However, its mechanism of action is still unclear. It is a calcium antagonist that affects most calcium channels, including those in vascular smooth muscle, and inhibits the release of calcium from the sarcoplasmic reticulum. Smooth muscle contraction is reduced by the deactivation of myosin light chain kinase activity. Vasodilation occurs and is responsible for magnesium’s beneficial properties. It also decreases the levels of circulating angiotensin-converting enzyme (ACE), leading to less endothelial activation and less vasopressin production, with a subsequent antihypertensive effect [11, 12]. The combination of peripheral antihypertensive effects with the cerebral vasodilator effect could explain the prophylactic anticonvulsive action of MgSO4 [13].

Labetalol is a nonselective, competitive β-adrenergic blocker and a selective, competitive α1-adrenergic blocker (ratio of blockade 7:1 respectively). It produces a rapid dose-dependent decrease in blood pressure without causing reflex tachycardia or a significant reduction in heart rate. Furthermore, labetalol has many important non-antihypertensive effects that may be beneficial in preeclampsia. It has an antiplatelet aggregation action, a thromboxane-reducing effect, and a fetal lung maturation-accelerating influence [13], and currently, it is recommended as the first-line treatment for blood pressure control in preeclampsia by the American College of Obstetricians and Gynecologists (ACOG) [1]. Labetalol was found to effectively reduce CPP without significantly affecting the middle cerebral artery velocities that might limit cerebral over perfusion in preeclampsia [7].

Several previous TCD studies reported an increase in CPP, and growing evidence supports cerebral over perfusion theory and subsequent cerebral edema as a leading theory for cerebral injuries in preeclampsia patients with severe features [7, 14–16], which comprises an attractive therapeutic target in these patients. Thus, by using TCD to measure middle cerebral artery blood flow indices and to estimate CPP, we compared the cerebral hemodynamic effects of both magnesium sulfate and labetalol medications which are commonly used in our preeclampsia patients assuming that using labetalol might be as effective or more as MgSO4 in reducing CPP.

## Methods

### Study design and population

This prospective randomized comparative clinical study was conducted in the surgical intensive care unit; Zagazig University Hospitals between October 2020 to February 2022 on singleton pregnant women who suffered from late-onset preeclampsia with severe features and aged 21–45 years old with a body mass index ≤ of 35 kg/m^2^. Informed written consent was obtained before study enrollment from the parturients or their legal guardian. The institutional review board of the Faculty of Medicine, Zagazig University approved this trial with the reference number (ZU-IRB#: 6353-23-3-2020) and it was registered under clinicaltrials.gov (NCT04539379).

Parturients who were diagnosed to have mild preeclampsia and those with a history of preexisting heart disease, atrial fibrillation or any rhythm abnormality, pulmonary disorders, allergy, or contraindications to either magnesium sulfate or labetalol and exposure to any of these study medications within 24 hours before the study enrollment as well as those with inadequate temporal window were excluded from this study.

Before study enrollment, all parturients were subjected to history taking, clinical examination, and investigated by complete blood picture, coagulation profile, liver functions test, and kidney functions test with the full examination by an obstetrician not involved in this study to diagnose those with severe preeclampsia who were immediately admitted and managed in the surgical intensive care unit. On physical examination, special attention was given to document vital signs (heart rate, systolic and diastolic invasive blood pressure); cardiac and chest condition and exclude contraindications to the study medications. The goal and endpoints of the study were discussed, to clarify the advantages and possible side effects of the study medications.

Preeclampsia was diagnosed according to the American College of Obstetricians and Gynecologists (ACOG) criteria defining preeclampsia as persistent elevation of systolic blood pressure (SBP) > 140mmHg and/or diastolic blood pressure (DBP) > 90mmHg after 20 weeks of gestation with significant proteinuria and preeclampsia with severe features was diagnosed depending on the presence of one or more of the following: Systolic blood pressure elevation ≥ 160mmHg or diastolic blood pressure ≥ 110mmHg measured on two occasions at least 4 hours apart, presence of pulmonary edema, decrease in the platelet count less than 100,000^*^10^9^/L, liver enzymes elevation to twice the upper limit normal concentration with severe persistent right upper quadrant or epigastric pain not attributed to another diagnosis and not responding to medications, doubling of the serum creatinine concentration or its increase more than 1.1mg/dl in the absence of other renal disease, visual disturbances, and/or headache unresponsive to medications [1].

Following ICU admission and preeclampsia severity features confirmation, all parturients fulfilling the inclusion criteria were subjected to baseline TCD evaluation and then randomly allocated into 2 equal groups to receive one of the study medications.

Randomization was performed by sealed opaque envelopes containing random numbers generated by an online application (https://www.randomizer.org/).

#### Magnesium sulfate group [group M]

The patients were given intravenous magnesium sulfate at a dose of 4gm intravenously over 20min as a loading dose then MgSO4 intravenous infusion was continued at a rate of 1gm/h for 24h or until obtain and stabilize the targeted blood pressure.

#### Labetalol group [group L]

The patients were given intravenous labetalol (Trandate™) available in 20mL ampoules containing 100mg labetalol (5mg/mL). Starting the infusion with 20mg/h and then titrate to obtain and stabilize the targeted blood pressure by adjusting the infusion as required every 15–30min to a maximum dose of 160mg/hr. When clinically appropriate after stabilization of blood pressure it was discontinued by weaning over 1–2 hours.

The target blood pressure in the two groups was SBP between 110 and 140mmHg, and DBP between 60 and 90mmHg maintained for at least 4 readings within 60 min.

Patients in both groups with persistent hypertension were treated with continuous intravenous infusion of 5 µg/min nitroglycerine with increasing the dose of 5 µg/min every 5 min until reaching the therapeutic goal.

Patients in the labetalol group who convulse were treated with MgSO4 with a loading dose of 4gm intravenously over 20min then MgSO4 intravenous infusion was continued at a rate of 1gm/h for 24h and if convulsion occurs in the MgSO4 group, Patients were continued to receive MgSO4. Other management of all patients were according to the standard protocol in the ICU.

Measured TCD parameters including mean flow velocity (cm/s), mean end-diastolic velocity (DIAS), and pulsatility index (PI) as well as the calculated cerebral perfusion pressure (CPP) and MCA velocity were recorded as basal measurements before administration of the study drugs then at post-treatment one and six hours after drug administration.

### Performance of the transcranial doppler

Transcranial doppler assessment was performed between the time of documented severe preeclampsia and study medication administration as a baseline measurement and then it was repeated post-treatment at one and six hours after drug administration.

All subjects were rest in left lateral position (15° left lateral recumbency) then after contact gel application, the 1–5 MHz ultrasound probe of Siemens Acuson X300 machine was placed between the ear and lateral orbital margin above the zygomatic bone on the temporal squama (the temporal window) to identify the middle cerebral artery (MCA). Measurements were performed bilaterally whenever possible, and the average value was calculated and recorded. If only one vessel is insonated, this value was used. All the measurements were taken by the same physician who is experienced in neurosonology and blinded to the study purpose.

The middle cerebral artery TCD measurements were the mean velocity (MV), the mean end-diastolic velocity (DIAS), and the pulsatility index (PI). The cerebral perfusion pressure (CPP) was estimated using the equation described by Aaslid et al which was validated in pregnant females [14]. CPP = [Mean Velocity / (Mean Velocity – Diastolic Velocity)] X (Mean Blood Pressure – Diastolic Blood Pressure) [14].

Middle cerebral artery velocity (MCA velocity) which is the middle cerebral blood flow component of the CPP formula (mean velocity / (mean velocity – diastolic velocity) was extracted and recorded to examine the impact of cerebral blood flow versus peripheral blood pressure on CPP changes.

### Data collection

The following data were collected for each patient: Age, Body mass index (BMI), and gestational age. Basal blood pressure and heart rate were assessed then monitored continuously and it was recorded at the time of each TCD measurement.

TCD of MCA parameters including the mean velocity (MV), the mean end-diastolic velocity (DIAS), and the pulsatility index (PI) as well as the calculated cerebral perfusion pressure (CPP) and MCA velocity were recorded as basal measurements before administration of the study drugs then at post-treatment one and six hours after drug administration.

The need for other antihypertensive drugs e.g., nifedipine, nitroglycerine, hydralazine was recorded for each group.

The occurrence of seizures in each group was monitored and managed. Also, any adverse effects of the study drugs such as hypotension (A systolic blood pressure of less than 90mmHg or diastolic of less than 60 mmHg), bradycardia (resting heart rate of under 60 beats per minute), persistent hypertension (Patients with persistent blood pressure above the target blood pressure), nausea and vomiting were noted and recorded.

### Sample size calculation

Using the (open Epi) program, the sample size was calculated to be 60 cases divided into 2 equal groups, (30 subjects) in each group. This was done based on assuming that the middle cerebral artery means velocity before and after labetalol administration in preeclampsia patients was 65 ± 10 versus 64 ± 12cm/s [7] at a confidence interval of 95% and power of test 80%.

### Statistical analysis

The Collected data were statistically analyzed using Statistical Package for Social, Science software (version 20, SPSS Inc., Chicago, IL). Continuous variables with a normal distribution were reported as mean ± SD and range. Categorical variables were summarized as frequencies and percentages. Numerical data were evaluated using independent T-test, repeated measures analysis of variance (ANOVA), and Least Significant Difference (LSD) was used to detect a significant difference between every 2 separate groups, while qualitative data were evaluated by Chi-square test (χ2). P values < 0.05 and < 0.001 were considered statistically significant and highly statistically significant respectively.

## Results

**Fig. 1:**
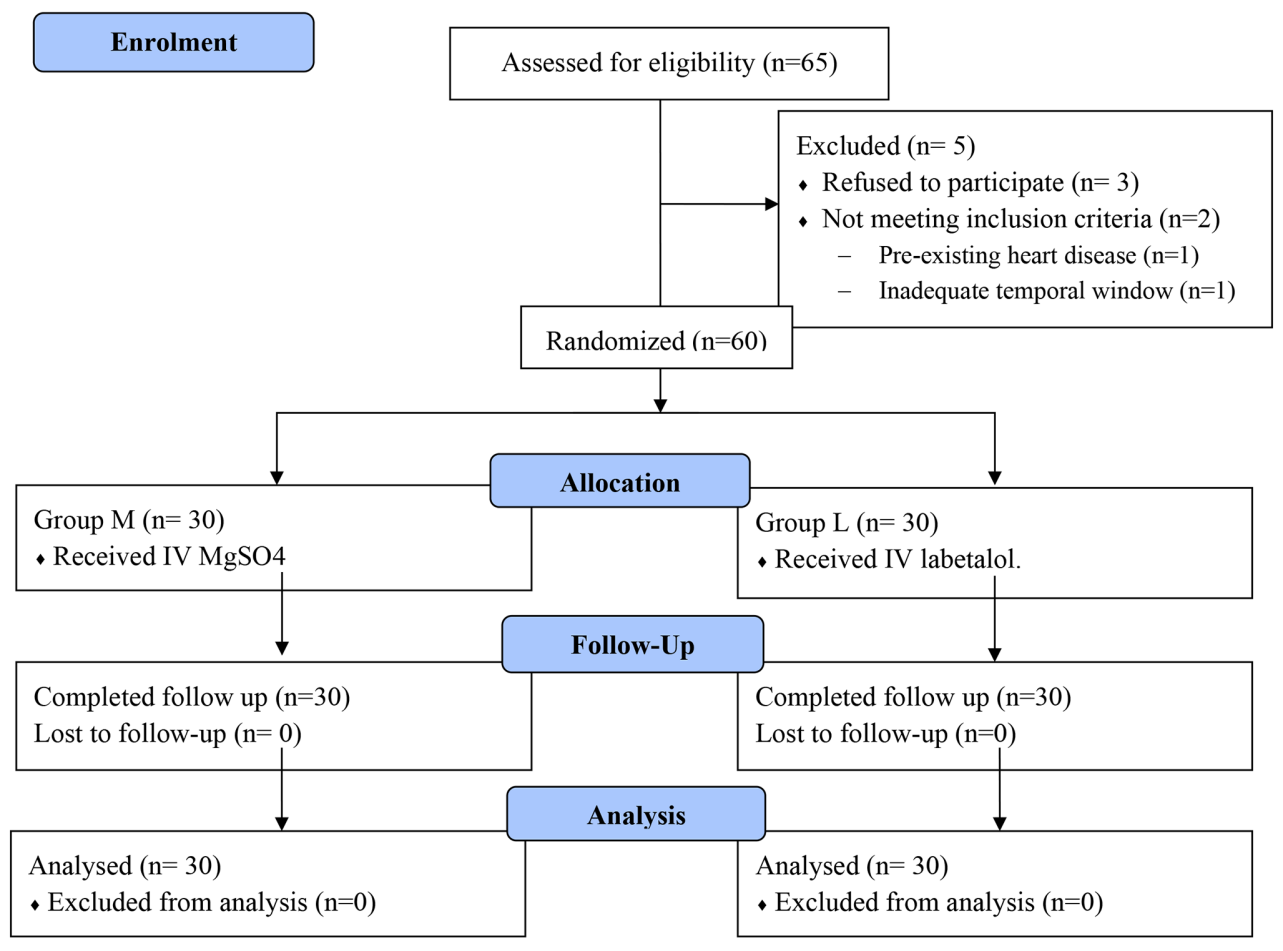
Study flow diagram n = number of patients, IV = Intravenous, MgSO4 = Magnesium sulfate

Sixty-five pregnant females suffered from preeclampsia were eligible for enrollment during our study period. From them 5 patients were excluded (three patients refused to participate in the study, one patient had preexisting heart disease, and one patient showed inadequate temporal window). Thus, sixty consented singleton pregnant females who suffered preeclampsia with severe features were included and randomly allocated into two equal groups (Fig. 1). The patients’ clinical characteristics (age, BMI, and gestational age), preeclampsia severity features, as well as the baseline laboratory data on admission, showed no statistically significant difference between the two studied groups. The mean age of included patients was 27.5 ± 4.62 years old in group M and 27.4 ± 4.70 years old in group L (p value = 0.891). All enrolled patients were suffering from late-onset preeclampsia with the gestational age of 34.8 ± 2.07 versus 34.5 ± 1.35weeks in group M and L respectively (p value = 0.608). All subjects were diagnosed to have severe preeclampsia depending on the presence of one or more of the severity features. Hypertension with persistent elevation of SBP ≥ 160 mmHg or DBP ≥ 110 mmHg was the commonest severity feature and it was found in all enrolled subjects (Table 1).

**Table 1:** Patients’ characteristics, preeclampsia severity features and laboratory results on admission of the studied groups

Characteristics		Group M (n = 30)	Group L (n = 30)	P value
**Age (years)**		27.5 ± 4.62	27.4 ± 4.70	0.891
**BMI (kg/m** ^**2**^ **)**		26.1 ± 2.30	26.2 ± 2.42	0.828
**Gestational age (weeks)**		34.8 ± 2.07	34.5 ± 1.35	0.608
**Preeclampsia severity features**				
Hypertension*	**Number (%)**	30 (100%)	30 (100%)	1
**Liver impairment**	**Number (%)**	4 (13.3%)	7 (23.3%)	0.317
**Renal impairment**	**Number (%)**	0 (0%)	2 (6.7%)	0.150
**Pul. edema**	**Number (%)**	0 (0%)	1 (3.3%)	0.313
**Thrombocytopenia**	**Number (%)**	2 (6.7%)	3 (10%)	0.640
**Visual disturbances**	**Number (%)**	4 (13.3%)	3 (10%)	0.688
**New onset headache**	**Number (%)**	4 (13.3%)	3 (10%)	0.688
**Laboratory data**				
**Hb (gm/dL)**		10.2 ± 1.04	10.4 ± 0.73	0.357
**Platelet (x10^** ^**3**^ **/mm** ^**3**^ **)**		179.1 ± 48.8	189.2 ± 51.8	0.442
**Albumin (gm/dL)**		3.32 ± 0.41	3.22 ± 0.35	0.301
**Creatinine (mg/dL)**		0.82 ± 0.26	0.85 ± 0.37	0.661
**AST (U/L)**		32.6 ± 9.5	36.7 ± 10.5	0.117
**ALT (U/L)**		36.2 ± 8.6	40.1 ± 10.2	0.116

Group M = Magnesium sulfate group, Group L = Labetalol group, n = Total number of subjects in each group, BMI = Body mass index, Pul.edema = pulmonary edema, Hb = hemoglobin, AST = aspartate aminotransferase, ALT = Alanine aminotransferase.

*Hypertension as a severity feature was recorded if systolic blood pressure (SBP) ≥ 160 mmHg or diastolic blood pressure (DBP) ≥ 110 mmHg measured on two occasions at least 4 hours apart.

Data were expressed as mean ± SD; number (percentage).

Independent samples student’s t-test.

P < 0.05 is significant.

The middle cerebral artery TCD measurements including the MV, DIAS, PI as well as the calculated CPP and MCA velocity showed no statistically significant difference between both groups at all times of measurements (Table 2). While comparing these measurements with the baseline reading in each group, both PI and calculated CPP were highly significantly decreased at 1 and 6 hours after either labetalol or magnesium sulfate administration in each group (Table 3). For group M PI was 0.77 ± 0.04 at baseline versus 0.66 ± 0.05 at 1hour and 0.66 ± 0.05 at 6 hours after MgSO4 administration (p value < 0.001) also the calculated CPP was significantly decreased from 103.3 ± 12.7mmHg to 87.8 ± 10.6mmHg and 89.8 ± 10.9mmHg (p value < 0.001) at 1 and 6 hours respectively. Similarly, in group L the PI was significantly decreased from 0.77 ± 0.05 at baseline before labetalol administration to 0.67 ± 0.05 and 0.67 ± 0.06 at 1 and 6 hours (p value < 0.001) after drug administration. Moreover, the calculated CPP was significantly decreased from 103.6 ± 12.6 mmHg to 86.2 ± 13.02mmHg at 1 hour and to 83.7 ± 14.6mmHg at 6 hours after labetalol administration (p value < 0.001).

**Table 2:** TCD measurements of the two studied groups at different timings

Variables	Group M (n = 30)	Group L (n = 30)	P-value
**MV (cm/s)**			
**Baseline**	68.1 ± 7.09	66.1 ± 6.52	0.272
**After 1 hour**	66.4 ± 6.92	63.3 ± 5.98	0.060
**After 6 hours**	67.1 ± 7.05	64.3 ± 6.78	0.117
**DIAS (cm/s)**			
**Baseline**	48.4 ± 5.42	46.8 ± 3.52	0.192
**After 1 hour**	46.9 ± 8.50	43.5 ± 7.50	0.108
**After 6 hours**	47.7 ± 6.32	44.5 ± 6.81	0.065
**PI**			
**Baseline**	0.77 ± 0.04	0.77 ± 0.05	0.712
**After 1 hour**	0.66 ± 0.05	0.67 ± 0.05	0.535
**After 6 hours**	0.66 ± 0.05	0.67 ± 0.06	0.306
**CPP (mmHg)**			
**Baseline**	103.3 ± 12.7	103.6 ± 12.6	0.856
**After 1 hour**	87.8 ± 10.6	86.2 ± 13.02	0.619
**After 6 hours**	89.8 ± 10.9	83.7 ± 14.6	0.074
**MCA velocity (cm/s)**			
**Baseline**	3.54 ± 0.44	3.62 ± 0.44	0.489
**After 1 hour**	3.47 ± 0.38	3.51 ± 0.35	0.657
**After 6 hours**	3.42 ± 0.30	3.54 ± 0.36	0.182

Group M = Magnesium sulfate group, Group L = Labetalol group, n = Total number of subjects in each group, MV = Mean Velocity, DIAS = Mean End Diastolic Velocity, PI = Pulsatility Index, CPP = Cerebral Perfusion Pressure, MCA Velocity = Middle Cerebral Artery Velocity.

Data were expressed as mean ± SD.

Independent samples student’s t-test.

P < 0.05 is significant.

**Table 3:** TCD measurements at different timings within each group

Variables	Baseline	After 1hour	After 6hours	P value
**Group M**				
**MV (cm/s)**	68.1 ± 7.09	66.4 ± 6.92	67.1 ± 7.05	0.373
**DIAS (cm/s)**	48.4 ± 5.42	46.9 ± 8.50	47.7 ± 6.32	0.470
**PI**	0.77 ± 0.04	0.66 ± 0.05*	0.66 ± 0.05	< 0.001
**CPP (mmHg)**	103.3 ± 12.7	87.8 ± 10.6*	89.8 ± 10.9	< 0.001
**MCA Velocity (cm/s)**	3.54 ± 0.44	3.47 ± 0.38	3.42 ± 0.30	0.285
**Group L**				
**MV (cm/s)**	66.1 ± 6.52	63.3 ± 5.98	64.3 ± 6.78	0.154
**DIAS (cm/s)**	46.8 ± 3.52	43.5 ± 7.50	44.5 ± 6.81	0.113
**PI**	0.77 ± 0.05	0.67 ± 0.05*	0.67 ± 0.06	< 0.001
**CPP (mmHg)**	103.6 ± 12.6	86.2 ± 13.02*	83.7 ± 14.6	< 0.001
**MCA Velocity (cm/s)**	3.62 ± 0.44	3.51 ± 0.35	3.54 ± 0.36	0.371

Group M = Magnesium sulfate group, Group L = Labetalol group, n = Total number of subjects in each group, MV = Mean Velocity, DIAS = Mean End Diastolic Velocity, PI = Pulsatility Index, CPP = Cerebral Perfusion Pressure, MCA Velocity = Middle Cerebral Artery Velocity.

Data were expressed as mean ± SD.

Repeated measure ANOVA test

P < 0.05 is significant.

* Both PI and CPP were statistically high significantly lower in both groups after 1 and 6 hours of drug administration compared to the baseline measurement.

**Fig. 2:**
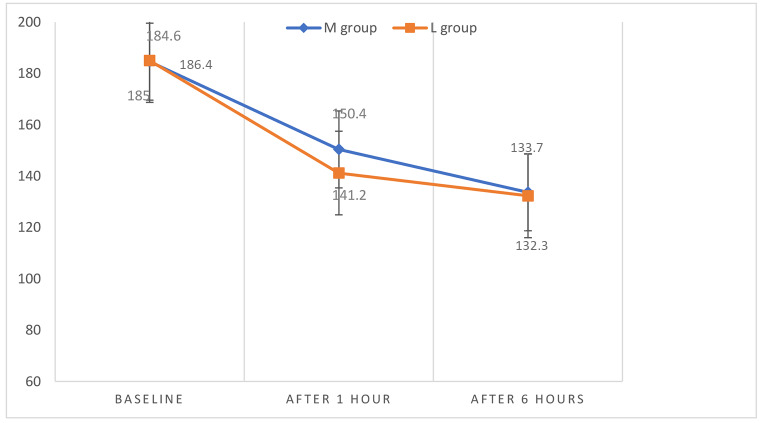
Systolic blood pressure measurements at different timings between the studied groups. M group = Magnesium sulfate group, L group = Labetalol group

**Fig. 3:**
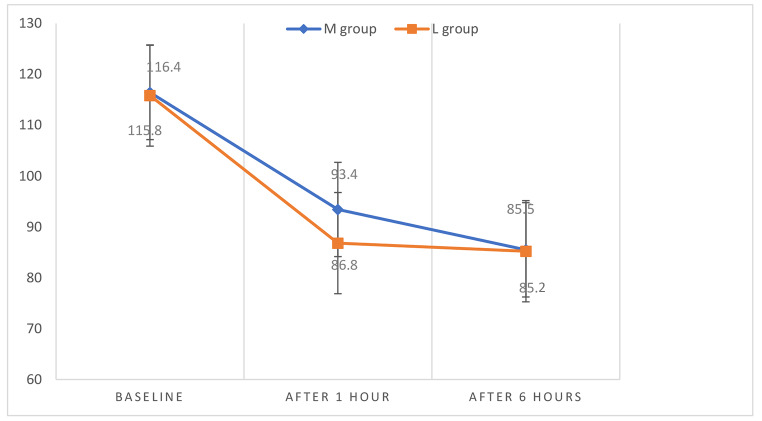
Diastolic blood pressure measurements at different timings between the studied groups. M group = Magnesium sulfate group, L group = Labetalol group

**Fig. 4:**
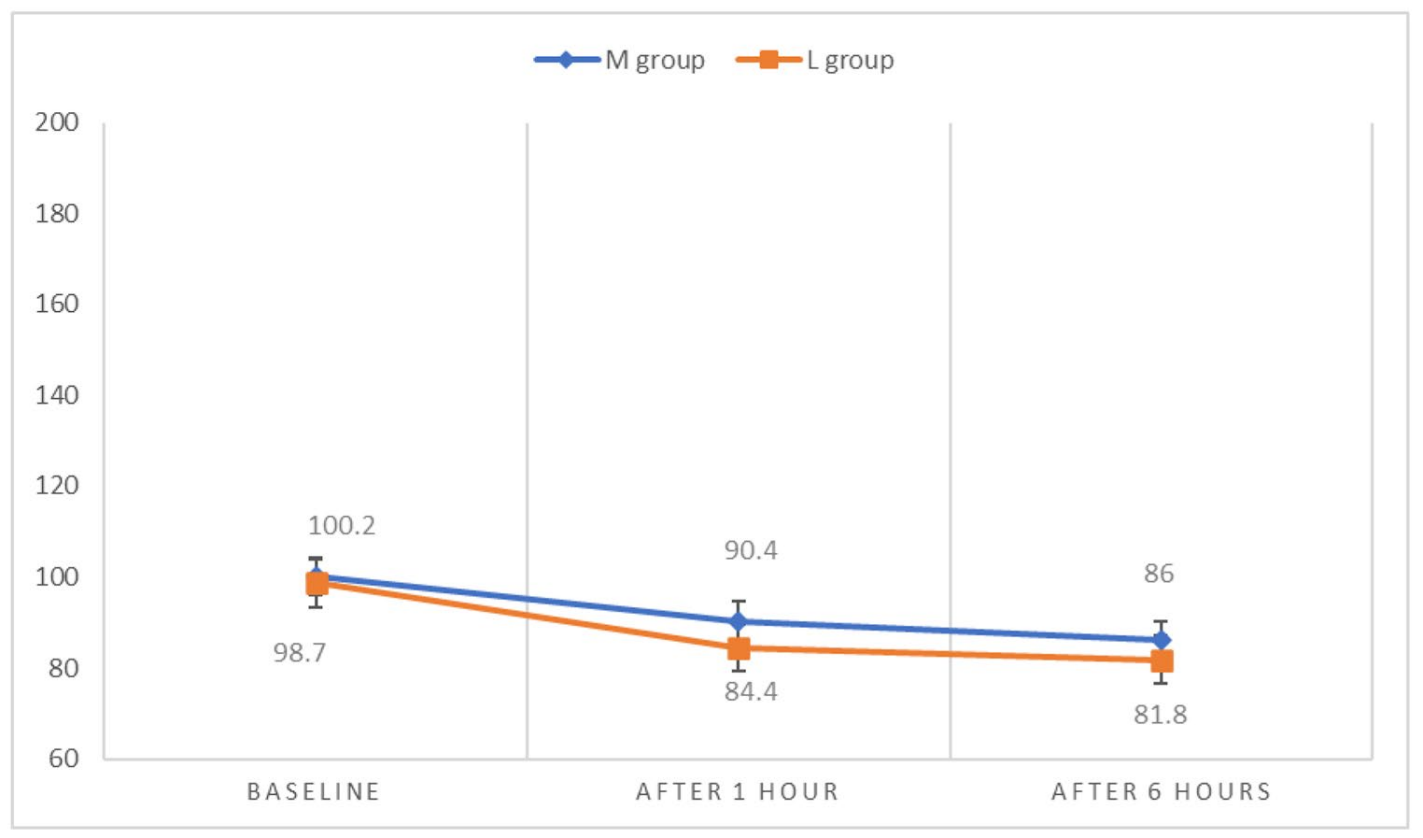
Heart rate measurements at different timings between the studied groups M group = Magnesium sulfate group, L group = Labetalol group

There was a statistically significant difference between the two studied groups regarding systolic, diastolic blood pressure, and heart rate after 1 hour of drug administration as it was found to be significantly higher among group M compared to group L with no statistically significant difference observed at other times of measurements between the two groups (Fig. 2, 3, 4). However, the systolic and diastolic blood pressures, as well as the heart rate, were found to be statistically significantly lowered in both groups after 1 and 6 hours of drug administration compared to baseline measurements. (Table 4)

**Table 4:** Hemodynamic measurements at different timings within each group

Variables	Baseline	After 1hour	After 6hours	P-value
**Group M**				
**SBP (mmHg)**	186.4 ± 16.5	150.4 ± 11.8*	133.7 ± 7.37*	< 0.001
**DBP (mmHg)**	116.4 ± 4.98	93.4 ± 6.03*	85.5 ± 6.51*	< 0.001
**HR (beat/min)**	100.2 ± 11.7	90.4 ± 10.1*	86 ± 8.49*	< 0.001
**Group L**				
**SBP (mmHg)**	185 ± 16.2	141.2 ± 10*	132.3 ± 7.79*	< 0.001
**DBP (mmHg)**	115.8 ± 4.65	86.8 ± 7.66*	85.2 ± 5.18*	< 0.001
**HR (beat/min)**	98.7 ± 9.63	84.4 ± 12.1*	81.8 ± 9.67*	< 0.001

Group M = Magnesium sulfate group, Group L = Labetalol group, n = Total number of subjects in each group,, SBP = Systolic blood pressure, DBP = Diastolic blood pressure, HR = Heart rate.

Data were expressed as mean ± SD.

Repeated measure ANOVA test

P < 0.05 is significant.

*SBP, DBP, and HR were significantly lower in both studied groups after 1 and 6 hours of drug administration compared to baseline readings.

The persistence of hypertension and the need to add another antihypertensive drug with the studied medication was statistically significantly higher in the magnesium group compared to the labetalol group with the other adverse effects showed no statistically significant difference in both groups. No statistically significant difference was found between both groups regarding seizures occurrence, seizures occurred in 7 cases in the labetalol group versus 4 cases in magnesium groups (p-value = 0.317) (Table 5).

**Table 5:** Adverse effects of the two studied drugs in both groups

Variables		Group M (n = 30 )	Group L (n = 30 )	P value
**Seizures**	**Number (%)**	4 (13.3%)	7 (23.3%)	0.317
**Need to add another antihypertensive**				
**drug**	**Number (%)**	23 (76.7%)	5 (16.7%)*	< 0.001
**Hypotension**	**Number (%)**	0 (0%)	1 (3.3%)	0.313
**Bradycardia**	**Number (%)**	0 (0%)	2 (6.7%)	0.150
**Nausea**	**Number (%)**	7 (23.3%)	5 (16.7%)	0.519
**Vomiting**	**Number (%)**	3 (10%)	2 (6.7%)	0.640

Group M = Magnesium sulfate group, Group L = Labetalol group, n = Total number of subjects in each group.

Data were expressed as numbers (percentage).

Independent samples student’s t-test.

P < 0.05 is significant.

* Persistence of hypertension and the need for another antihypertensive drug were highly significantly lower in the labetalol group.

## Discussion

The most common cause of maternal mortality in preeclampsia is neurological complications. Hence, it is essential to understand the underlying pathophysiological mechanisms of these cerebral complications. Alteration of cerebral autoregulation and CPP in preeclampsia is a leading theory [17]. Several TCD studies revealed an increase in CPP in preeclampsia, and growing evidence supports cerebral barotrauma and over perfusion theory and subsequent cerebral edema [7, 14–16] which comprises an attractive therapeutic target to prevent cerebral injuries and seizures in severe preeclampsia.

Our study evaluated magnesium sulfate and labetalol medications, which are commonly used in our preeclampsia patients, with the CPP and CBFV endpoints by using the TCD.

In the current study, both MgSO4 and labetalol were found to decrease CPP significantly at 1 and 6h after administration, with no significant change in the middle cerebral artery blood flow indices (MV, DIAS, and MCA velocity) indicating that the CPP reduction is due to the decrease in the blood pressure and not due to change in CBFV. Additionally, PI was found to be significantly decreased at 1 and 6h after administration of either MgSO4 or labetalol. However, the extent of CPP reduction and the blood flow indices did not differ significantly when labetalol was compared to MgSO4.

Currently, severe preeclampsia and eclampsia are treated with magnesium sulfate [1] and it is considered the drug of choice for seizure prevention in parturients with preeclampsia [1, 18]. Although its effectiveness in the prevention and treatment of eclampsia is well known, its mechanism of action is unclear, with several mechanisms proposed, including its actions as a central anticonvulsant at the N-methyl-D-aspartate receptor, anti-inflammatory effects with inhibition of neuroinflammation, and protection of the blood-brain barrier to reduce cerebral edema formation [19].Moreover, magnesium sulfate is a calcium antagonist that acts on calcium channels in vascular smooth muscle, resulting in vasodilatation [11, 12] with some studies suggesting a concentration-dependent vasodilator effect of magnesium sulfate in both cerebral and mesenteric arteries [20].

However, our findings of no significant change in MCA blood flow indices after magnesium sulfate administration question its role as a cerebral vasodilator. In accordance with our findings, Hatab et al. used a velocity-encoded phase-contrast magnetic resonance imaging technique and found no change in cerebral blood flow or the diameters of the middle and posterior cerebral arteries after 6-gram magnesium sulfate loading dose infusion in preeclampsia patients [21]. In addition, several previous studies that used TCD in evaluating MgSO4 effects in preeclampsia have reported that magnesium sulfate did not change middle cerebral artery velocity and the diameter of large cerebral arteries [15, 22]. Also, in healthy volunteers, Sherman et al., in their study found that magnesium sulfate infusion has no effect on the middle cerebral artery blood flow velocity, cerebral autoregulation, and cerebral vascular reactivity to carbon dioxide [23].

The pulsatility index has been recognized to have a linear relationship with the intracranial pressure (ICP) and it is considered as an indirect estimate of ICP. Also, PI was reported to represent intracranial compliance and distal cerebral vascular resistance [24]. In the current study, there was a significant decrease in the PI after MgSO4 administration with the absence of any significant change in MCA blood flow indices suggests that the cerebral vasodilatory effects of MgSO4 may be on the distal vessels and not the large cerebral arteries. This is in line with Belfort et al. who evaluated the effect of 6 gm magnesium sulfate intravenous bolus on the maternal cerebral blood flow velocity in 12 preeclampsia patients and concluded that magnesium sulfate when compared to placebo markedly reduce the PI of the middle cerebral artery signifying its role as a vasodilator of the small-diameter vessels and relieving cerebral ischemia [25]. As well, Hatab et al did not find any statistically significant change either in the blood flow or the diameter of middle and posterior cerebral arteries after MgSO4 administration reporting that it may not vasodilate large cerebral vessels in preeclampsia [21]

Believing that CPP reduction might have a key role in the management of preeclampsia patients, our study findings could support this theory. Since MgSO4 administration decreased CPP while maintaining CBF in preeclampsia patients with severe features and elevated baseline CPP in our study,This discovery may also be a proposed mechanism of action for MgSO4 in seizure prevention.MgSO4 is well-known to obviously prevent eclamptic seizures and has been used for decades; it is currently recommended as the drug of choice for eclampsia prophylaxis [1].

The same decrease in CPP with no effect on CBF in severe preeclampsia patients was also found after labetalol administration in the current study. The previous studies evaluating the effects of antihypertensive drugs on maternal cerebral hemodynamics of preeclampsia patients are scarce [7, 15, 26, 27]. In line with our results, Belfort et al evaluated the middle cerebral arteries blood flow velocity of eight preeclampsia patients before and after 200mg oral labetalol administration. They reported a decrease of CPP with no effect on cerebral perfusion after 180 minutes [7]. As well, Tolcher et al compared CPP changes in preeclampsia patients before and after intravenous labetalol versus oral nifedipine administration reporting that labetalol has no effect on MCA velocity and that it may have no direct effect on cerebral vessels [28]. Additionally, they did not find any change in CPP after labetalol administration which is different from our results and could be attributed to the different timing of TCD evaluation after labetalol administration (30min in Tolcher et al study versus 60min in our study).

The findings of our study showed that the mean values of SBP, DBP, and HR were significantly lower in the labetalol group after 1 hour of drug administration compared to the MgSO4 group and that parturients in the MgSO4 group were more likely to require an additional antihypertensive drug to achieve blood pressure target. Regarding the occurrence of seizures, there were no statistical difference between the two groups, seizures in the labetalol group was reported in 7 cases (23.3%) versus 4 cases (13.3%) in the magnesium group. 2 patients in the labetalol group was found to have renal impairment and they were adequately managed with no seizures occurred and no need to add MgSO4 which is known to require frequent monitoring of its serum levels in renal impairment patients. Rates of other adverse effects did not differ significantly between labetalol and MgSO4 groups.

MgSO4 has been shown in previous studies to have an antihypertensive effect in patients with severe pregnancy-induced hypertension [29, 30]. However, this antihypertensive effect was not enough to control blood pressure in early-onset preeclampsia and in parturients aged 40 years or more in Takenaka et al, study [29]. Also, Belford et al, noted that hydralazine was required more frequently for controlling blood pressure in patients receiving MgSO4 than those receiving nimodipine [30]. On the other hand, several studies have reported satisfactory blood pressure control with labetalol administration in preeclampsia with both oral and intravenous administration [31, 32]. Adding to this, the use of oral labetalol for acute control of severe preeclampsia was demonstrated to be comparable with hydralazine [31].

Given the recognized role of cerebral autoregulation impairment and the hypertensive encephalopathy pathophysiological mechanism, and the fact that the challenge in severe preeclampsia patients is to reduce blood pressure and CPP without jeopardising CBF, our findings support the evidence of MgSO4’s ability to reduce CPP while maintaining CBF as a potential mechanism of action in severe preeclampsia patients with elevated baseline CPP.This CPP normalizing effect of MgSO4 was comparable with that of labetalol, which was found to reduce CPP with no effect on CBF in our enrolled patients. Therefore, labetalol, which has been used for many years in preeclampsia patients, is both effective and safe for controlling blood pressure and reducing CPP with no effect on CBF in these patients.

Using labetalol with no magnesium sulfate was supported by several previous studies. Walker in his experience report demonstrated that labetalol was the only antihypertensive drug used in over 500 preeclampsia patients with no MgSO4 administration and only one of these patients had a seizure that may be due to inadequate blood pressure control [32]. Also, Warren et al, in their study compared labetalol versus MgSO4 administration in three hundred twenty-two preeclampsia patients randomized into two groups (147 patients received MgSO4 and 175 patients received labetalol). They reported only two patients in each group developed seizures (labetalol 1.1% vs MgSO4 1.4%) and that patients in the MgSO4 group required additional antihypertensive drugs more significantly than those in the labetalol group [33].

One of the main strengths of our study is that its prospective randomized comparative design comparing the cerebral hemodynamic effects of the two most commonly used drugs in preeclampsia patients. We suppose that the presented cerebral hemodynamic data add to the increasing evidence supporting the cerebral over perfusion pathophysiologic mechanism of cerebral injuries in preeclampsia with severe features.

It is interesting to note that our study has several limitations including the small sample size that clearly is insufficient to address the safety issues. However, we believe that labetalol which has been used for many years is both effective and safe for controlling hypertension in preeclampsia. Secondly, the difficulty of accurately predicting preeclampsia patients with elevated CPP based on blood pressure alone. Although the CPP and blood pressure are obviously linked, they are not directly correlated in preeclampsia as abnormal cerebral autoregulation plays a vital role [34]. Moreover, the presence of headache that may be associated with elevated CPP should not be used clinically as a screening sign since there is a considerable proportion of patients with elevated CPP did not have a headache in Tolcher et al study [28]. Also, in the present study headache was recorded in a small proportion of our patients. Therefore, to identify those with high CPP, we used TCD to provide a non-invasive and rapid assessment of CBF and to estimate CPP. Consequently, we could recognize patients who would benefit from therapeutic strategies to normalize CPP assuming that this TCD clinical application would be beneficial in severe preeclampsia and imminent eclampsia patients. Lastly, the effects of the studied drugs on pregnancy and neonatal outcomes should be studied in the future.

## Conclusion

In conclusion, in preeclampsia patients with severe features both magnesium sulfate and labetalol reduce CPP while maintaining the CBF and this labetalol CPP reducing effect is as effective as magnesium sulfate. It is worth mentioning that our findings should be considered preliminary and further large multicenter studies are required to verify these results.

## Data Availability

The data are available from the corresponding author on reasonable request.

## Abbreviations

CPP: Cerebral perfusion pressure
MgSO4: Magnesium sulfate
TCD: Transcranial doppler
MCA: Middle cerebral artery
PI: pulsatility index
CNS: Central nervous system
ACOG: American College of Obstetricians and Gynecologists
SBP: Systolic blood pressure
DBP: Diastolic blood pressure
MV: Mean velocity the
DIAS: mean end-diastolic velocity
